# P-852. Impact of Social Determinants of Health on Synchronous and Asynchronous Antibiotic Prescribing Practices: Quality Improvement Experience of a Large Primary Care Network (PCN)

**DOI:** 10.1093/ofid/ofaf695.1060

**Published:** 2026-01-11

**Authors:** Mike Sportiello, Jineane V Venci, Robert J Fortuna, Alexandra Yamshchikov

**Affiliations:** Emory University, Atlanta, GA; University of Rochester Medical Center, Rochester, New York; University of Rochester, Penfield, NY, New York; University of Rochester School of Medicine and Dentistry, Rochester, NY

## Abstract

**Background:**

Education and feedback interventions improve antibiotic prescribing practices in outpatient settings. Studies evaluating equitable impact of such interventions across patient groups and care modalities, such as telemedicine, are limited.

**Table 1.**
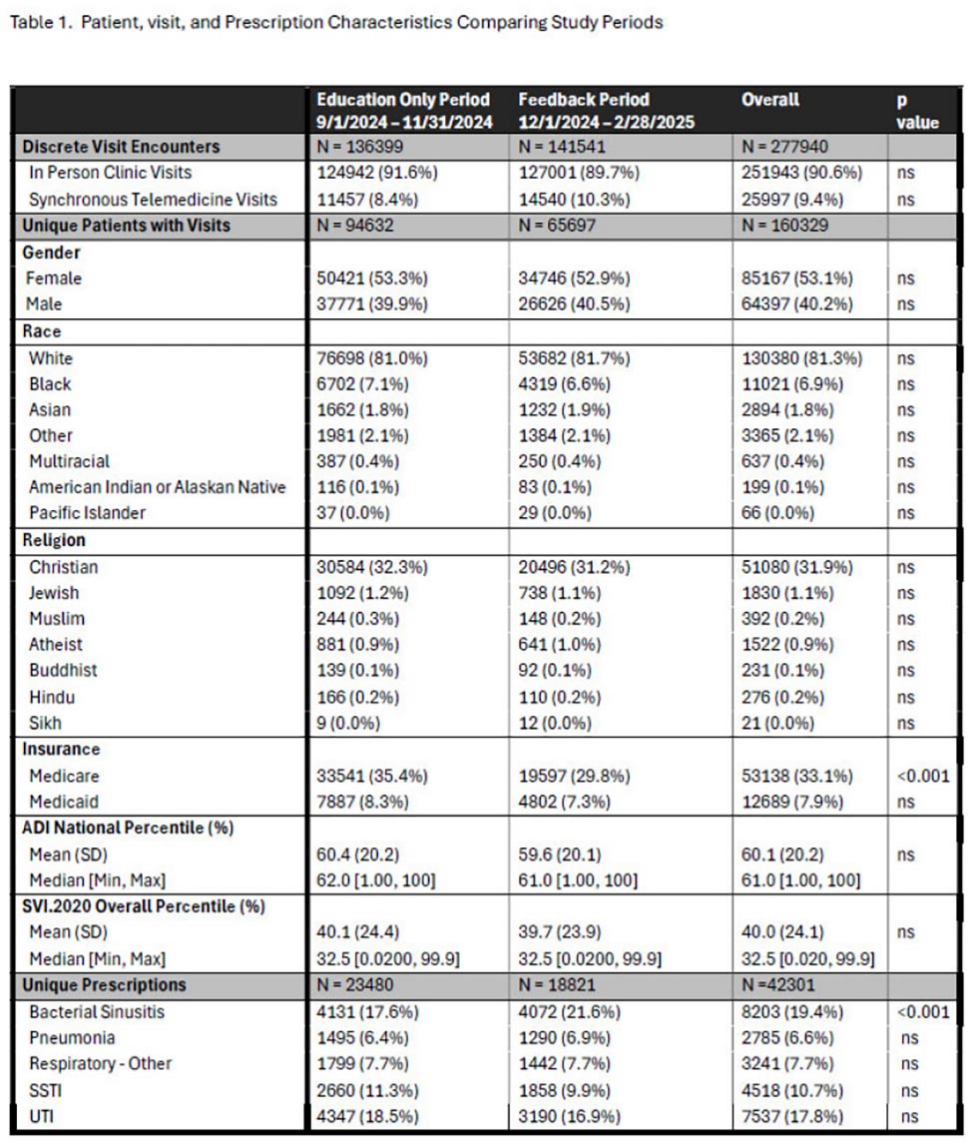
Patient, Visit, and Prescription Characteristics Comparing Study Periods

**Figure 1.**
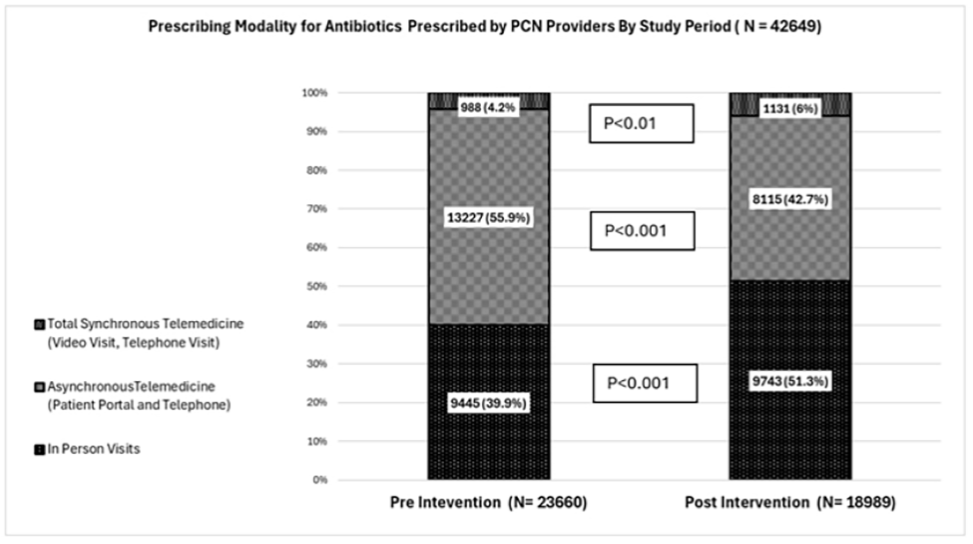
Prescribing Modality for Antibiotics Prescribed by PCN Providers by Study Periods

**Methods:**

Antibiotics prescriptions in a large PCN were extracted pre and post implementation of a QI feedback initiative focusing on antibiotic utilization. Total synchronous (clinic and synchronous telemedicine (ST)) visits were extracted. Antibiotic prescriptions from synchronous encounters were disaggregated by prescription mode (clinic or ST) and patient sociodemographic parameters, matched with appropriately disaggregated visit denominators, and prescription rates calculated. Antibiotics prescribed via asynchronous telemedicine (AST - patient portal/telephone) were evaluated independently. Analysis was performed with Χ2, Z test, and logistic regression in GraphPad and R v4.4.3.

**Results:**

Total 267,572 network registry patients incurred 277,940 synchronous visits and received 42,301 antibiotic prescriptions (Table 1), with reduction in overall antibiotic prescribing rate from 17.4 to 13.4 /100 visits (p < 0.000001) and shift away (Figure 1) from AST as most common prescribing modality post intervention (OR 0.69 [0.63-0.77], p< 0.000001). Men and Persons of Color (POC) were less likely to receive antibiotics during synchronous visits (OR 0.71 [0.65-0.78], p < 0.000001 and OR 0.54 [0.45-0.64] p < 0.000001), including clinic (OR .71 [0.64-0.77] p< 0.000001) and (OR 0.53 [0.44-0.64] p < 0.000001), respectively. Among patients receiving antibiotics, POC were more likely to be prescribed by telemedicine, but were still less likely to receive any antibiotic via ST (OR 0.59 [0.36-0.92], p=0.028) compared to other groups. Men (OR 0.6, [0.56-0.65] p< 0.000001), older than 65 (OR 0.6 [0.52-0.68] p< 0.000001), and Medicare patients (OR 0.78 [0.7-0.89] were less likely to access ST as a care modality.

**Conclusion:**

An educational intervention, combined with prescribing feedback, resulted in reduction of antibiotic rates and a shift away from asynchronous prescribing. Differences between antibiotic prescription rates and modalities exist based on race and sex. Further work must be done to investigate possible drivers of inequities.

**Disclosures:**

All Authors: No reported disclosures

